# LIMPRINT Study: The Turkish Experience

**DOI:** 10.1089/lrb.2019.0015

**Published:** 2019-04-17

**Authors:** Pinar Borman, Christine Moffatt, Susie Murray, Aysegul Yaman, Merve Denizli, Meltem Dalyan, Sibel Unsal-Delialioğlu, Sibel Eyigör, Figen Ayhan, Burcu Duyur Çakıt, Secil Vural, Oya Özdemir, Eda Kurt, Evrim Coşkun Çelik, Lale Cerrahoğlu, Müge Kepekçi, Fusun Terzioğlu, Ayşe Arikan Donmez

**Affiliations:** ^1^ Department of Physical Medicine and Rehabilitation (PMR), University of Hacettepe Faculty of Medicine, Ankara, Turkey.; ^2^ Lymphedema Research and Practice Center, Hacettepe University, Ankara, Turkey.; ^3^ Department of Nursing, School of Social Sciences, Nottingham Trent University, Nottingham, United Kingdom.; ^4^ CRICP, London, United Kingdom.; ^5^ Department of Physical Medicine and Rehabilitation (PMR), Ankara Physical Medicine and Rehabilitation Training and Research Hospital, Ankara, Turkey.; ^6^ Department of Physical Medicine and Rehabilitation (PMR), Faculty of Medicine, University of Ege, İzmir, Turkey.; ^7^ Department of Physical Medicine and Rehabilitation (PMR), Faculty of Medicine, University of Ahi Evran, Kırşehir, Turkey.; ^8^ Department of Physical Medicine and Rehabilitation (PMR), İstanbul Training and Research Hospital, İstanbul, Turkey.; ^9^ Department of Physical Medicine and Rehabilitation (PMR), Faculty of Medicine, University of Celal Bayar, Manisa, Turkey.; ^10^ Department of Physical Medicine and Rehabilitation (PMR), Kanuni Sultan Süleyman Training and Research Hospital, İstanbul, Turkey.; ^11^ Department of Nursing, Faculty of Health Science, Atılım University, Ankara, Turkey.; ^12^ Department of Internal Medicine Nursing, University of Hacettepe Faculty of Nursing, Ankara, Turkey.

**Keywords:** lymphedema, lymphoedema, chronic edema, LIMPRINT, quality of life, impact

## Abstract

***Background:*** Lymphedema and chronic edema is a major health care problem in both developed and nondeveloped countries The Lymphoedema Impact and Prevelance - International (LIMPRINT) study is an international health service-based study to determine the prevalence and functional impact in adult populations of member countries of the International Lymphoedema Framework (ILF).

***Methods and Results:*** A total of 1051 patients from eight centers in Turkey were recruited using the LIMPRINT study protocol. Data were collected using the core and module tools that assess the demographic and clinical properties as well as disability and quality of life (QoL). Most of the Turkish patients were recruited from specialist lymphedema services and were found to be women, housewives, and having secondary lymphedema because of cancer treatment. The duration of lymphedema was commonly <5 years and most of them had International Society of Lymphology (ISL) grade 2 lymphedema. Cellulitis, infection, and wounds were uncommon. The majority of patients did not get any treatment or advice before. Most of the patients had impaired QoL and decreased functionality, but psychological support was neglected. Although most had social health security access to lymphedema centers, nevertheless access seemed difficult because of distance and cost.

***Conclusion:*** The study has shown the current status and characteristics of lymphedema patients, treatment conditions, the unmet need for the diagnosis and treatment, as well as burden of the disease in both patients and families in Turkey. National health policies are needed for the prevention, diagnosis, and treatment in Turkey that utilize this informative data.

## Introduction

Lymphedema is an incurable, debilitating, and progressive condition, characterized by persistent swelling of one or more parts of the body, because of the impairments in lymph transport. This chronic and progressive disease can occur at any time after cancer surgeries, can arise from congenital malformation of the lymphatic system, or because by damage to lymphatic vessels.^1,2^ It is a major health care problem in both developed and nondeveloped countries. It is serious because of its long-term physical and psychosocial consequences for the patients, if left untreated. When lymphedema is not diagnosed and treated in the earlier stages, the prognosis for these patients is worse and treatments are more costly. Lymphedema frequently leads to physical, emotional, and psychological challenges and impairs the quality of life (QoL) if it is underrecognized and undertreated.^3,4^ Treatment cost of lymphedema has also been identified as a barrier. The support and funding of medical conditions and complex care needs will ease the stress and treatment burden associated with lymphedema.^5^ There are also challenges of managing complex lymphedema patients with obesity, and those associated with chronic medical conditions and wounds.^6^ Therefore the awareness of this chronic condition by both health professionals and patients—knowledge comprising the characteristics of lymphedema patients, difficulties finding appropriate treatments or funding for care, and the impact of disease on functional, psychosocial status, and QoL are of great importance, especially in developing countries.

Lymphedema has been a rising condition in Turkey over the past 10–12 years. Awareness about lymphedema was low and the treatment methods were unknown and certified lymphedema specialists were lacking until recent years.^7^ There are no data about the incidence of lymphedema in Turkey. Patient characteristics or experiences of some patients are reported in some small studies.^7–9^

The LIMPRINT^©^ study is an international multisite health service-based study to determine the prevalence and functional impact of lymphedema/chronic edema in the adult population of member countries of the International Lymphoedema Framework (ILF). It aims to estimate the proportion of patients with chronic edema and those with a concurrent wound.

Turkey has been a member of the ILF since 2017 under the auspices of the Anatolian/Turkish Lymphedema Association (ALA), but the LIMPRINT study was first noticed by Dr. Borman, Chair of the ALA at the ILF Meeting in Glasgow, 2014 (www.ilfconference.org). She was influenced by the presentations from other countries and was interested to find out how a study of Turkish lymphedema patients could be made and compare it with different countries. The participation in LIMPRINT study would be valuable and provide important information about the demographic, social, and QoL characteristics of Turkish patients. The study results would show the current status and characteristics of lymphedema patients, treatment conditions, the unmet need for diagnosis and treatment of those suffering with the condition, and burden of the disease in both patients and families in Turkey. The LIMPRINT study would also allow the comparison with different populations from different countries. In addition the data would be informative for developing national health polices and reimbursement procedures in diagnosis and treatment of lymphedema in Turkey.

## Materials and Methods

Considering all these points, an interest in being part of LIMPRINT was shared with the executive members of ALA, and after unanimous approval, a request for Turkey to be involved was officially made in 2014 and accepted by the ILF.

Two main institutions are related to lymphedema in Turkey: Anatolian Lymphedema Association and Hacettepe University Lymphedema Practice and Research Center. Therefore the participation in this international multicenter study proposal was sent to the health professional delegates of the ALA from different parts of Turkey working in centers who are managing patients with lymphedema. In addition, Hacettepe University Lymphedema Practice and Research Center actively engaged and recruited a great number of patients in collaboration with the Department of Physical Medicine and Rehabilitation. Vascular surgeons and nurses were also informed. Most of the surgeons were not interested in joining the study. After this initial information the proposal was sent to 10 centers with 8 accepting to be part of the study.

The local steering groups were Anatolian (Turkish) Lymphedema Association and Hacettepe University Lymphedema Practice and Research Center. The stakeholders were as follows, from five different areas of the country:

(1) University of Hacettepe Faculty of Medicine Department of Physical Medicine and Rehabilitation (PMR) and Hacettepe University Lymphedema Research and Practice Center (Dr. Pınar Borman, Dr. Merve Denizli, Dr. Ayşegül Yaman, Dr. Oya Özdemir, Dr. Fusun Terzioğlu, and Ayşe Arikan Dönmez).
(2) Ankara Rehabilitation Training and Research Hospital (Dr. Meltem Vural, Dr. Sibel Ünsal Delialioğlu).
(3) Ankara Training and Research Hospital Clinic of PMR (Dr. Figen Ayhan, Dr. Burcu Duyur Çakıt, Dr. Seçil Vural).
(4) Kirsehir Ahi Evran University Department of PMR (Dr. Eda Kurt).
(5) Ege University Medical Faculty Department of PMR, İzmir (Dr. Sibel Eyigör).
(6) Istanbul Rehabilitation Training and Research Hospital Clinic of PMR (Dr. Evrim Coşkun Çelik).
(7) Istanbul Kanuni Sultan Süleyman Education and Research Hospital (Dr. Muge Kepekçi).
(8) Manisa Celal Bayar University Medical Faculty Department of PMR (Lale Cerrahoğlu).

All centers gained approval from their local ethical committees. The coordinator of the Turkish study was the chair of ALA and director of the HU Lymphedema Practice and Research Center—P.B. All the LIMPRINT questionnaires were translated to Turkish and back translated to ensure accuracy of language. The QoL questionnaires lymphedema quality of life (LYMQOL)-arm and LYMQOL-leg^10^ did not have Turkish validation. The cross-cultural Turkish validation studies of the LYMQOL-arm and LYMQOL-leg questionnaires were performed before this study began adding further validity to the methods.^11,12^ Then the Turkish data collection forms were sent to the included centers. All the centers filled the questionnaires and sent them by ordinary mail to the coordinator and they were then returned when completed in batches of 30. Data entry was undertaken from one center (Hacettepe University) with each center given an individual code.

The patients were recruited to the study according to the inclusion and exclusion criteria of the LIMPRINT study protocol. Data were collected using a Core Tool to determine the prevalence of chronic edema and a set of five Module Tools to assess the impact of chronic edema on the lives of sufferers. Data were entered into a secure central on-line database. The core tools included questions about type of facility in which data are collected, demographics, level of obesity, mobility, relevant comorbidities, classification and history of lymphedema, cellulitis history, categories of treatment, site of swelling, wound area, access to treatment, and subjective control of swelling. The module tools comprised demographics and disability, QoL, details of swelling, wounds, and cancer. The Turkish version of World Health Organization Disability Assessment Schedule 2.0 (WHODAS 2.0) was used to assess disability,^13,14^ and LYMQOL^10–12^ and European Quality of Life Five Dimensional Questionnaire (EQ-5D)^15,16^ tools were used to assess QoL.

WHODAS 2.0 is a validated 12-item disability assessment schedule. It includes questions exploring the patient's personal circumstances for example, housing, employment, and education.^14,15^

LYMQOL is a validated condition-specific QoL assessment instrument (it is not validated for patients with lymphatic filariasis) that assesses the impact of lymphedema on the patient's everyday living and health-related QoL. There is a tool for patients with lymphedema of the upper limb and one for the lower limb.^11–13^

EQ-5D is a generic QoL instrument applicable to a wide range of health conditions and provides a simple descriptive profile and single index value for health status.^15^ EQ-5D is primarily intended for self-completion and is simple and quick to complete. Turkish validation has previously been made and used in this study.^16^

## Results

A total of 1051 patients from eight centers of five different geographical areas took part in the study. Most of the patients were recruited from specialist lymphedema services. The majority of patients were women, housewives, nonobese, had full range of movement, and walked independently. The most common comorbidity was diabetes followed by hypertension. Half the patients stated that their edema was not under control. The core demographic properties of the patients are given in Table 1.

**Table 1.** The Core Data of Turkish Patients Comprising the Demographic and Disease-Related Variables (*N* = 051)

	n *(%)*
Type of facility	
Acute inpatient	53 (5.04)
Acute outpatient	426 (40.53)
General practitioner	1 (0.10)
Nursing home	2 (0.19)
Specialist Lymphoedema Centre	569 (54.14)
Gender	
Female	980 (93.24)
Male	71 (6.76)
Age	
Mean (minimum, maximum)	53.42 (7–85)
Median	54
Age groups, years	
5–14	5 (0.48)
15–44	231 (21.98)
45–64	615 (58.52)
65–74	161 (15.32)
75–84	38 (3.62)
85 plus	1 (0.10)
Obesity	
Under weight	14 (1.33)
Normal weight	630 (59.94)
Obese	357 (33.97)
Morbidly obese	50 (4.76)
Lower limb mobility	
Bed bound	10 (0.95)
Chair bound	13 (1.24)
Walks with aid	71 (6.76)
Walks unaided	957 (91.06)
Upper limb mobility	
No function	3 (0.29)
Limited range of movement	138 (13.13)
Full range of movement	910 (86.58)
Comorbidity	
Diabetes mellitus	210 (19.98)
Heart failure/ischemic heart disease	84 (7.99)
Neurological disease	33 (3.14)
Peripheral arterial disease	115 (10.94)
None of these	711 (67.65)
Subjective control of swelling	
Yes	393 (48.70)
No	414 (51.30)

The classification of lymphedema was mostly secondary (85%) and caused by cancer (79%) with 85% suffering from breast cancer, followed by venous insufficiency, lipedema, immobility, and obesity. The cellulitis and infection or hospitalization for cellulitis and infection were infrequent. The duration of lymphedema was <5 years in majority of the patients. The whole group disease characteristics and distribution of variables according to gender are given in Table 2.

**Table 2.** The Turkish Edema Characteristics in the Core Tool (*N* = 051)

	*Total (*N = *1051)*	*Female (*n = *980)*	*Male (*n = *71)*	*χ^2^ (df) or* p*-value*
Classification				
Primary	152 (14.48)	129 (13.18)	23 (32.39)	19.75 (1)
Secondary	898 (85.52)	850 (86.82)	48 (67.61)	<0.001
Secondary swelling				
Cancer	717 (79.58)	698 (81.92)	19 (38.78)	53.09 (1)
Noncancer	184 (20.42)	154 (18.08)	30 (61.22)	<0.001
Cancer-related secondary LE				
Treatment related	715 (99.72)	696 (99.71)	19 (100.0)	0.99^a^
Metastatic	8 (1.12)	7 (1.00)	1 (5.26)	0.19^a^
Noncancer-related secondary LE				
Venous	92 (50.0)	71 (46.10)	21 (70.00)	0.017
Immobility	48 (26.09)	36 (23.38)	12 (40.00)	0.058
Obesity	84 (45.65)	74 (48.05)	10 (33.33)	0.14
Lymphatic filariasis	5 (2.72)	4 (2.6)	1 (33.3)	0.99^a^
Noncancer other	57 (30.98)	51 (33.12)	6 (20.00)	0.16
Duration				
<6 Months	273 (26.00)	260 (26.56)	13 (18.31)	
6 Months to 1 year	128 (12.19)	122 (12.46)	6 (8.45)	
1–2 Years	142 (13.52)	133 (13.59)	9 (12.68)	6.64 (5)
2–5 Years	219 (20.86)	201 (20.53)	18 (25.35)	0.25
5–10 Years	146 (13.90)	136 (13.89)	10 (14.08)	
10+ Years	142 (13.52)	127 (12.97)	15 (21.13)	
History of cellulitis				
Yes	232 (22.07)	206 (21.02)	26 (36.62)	9.37 (1)
No	819 (77.93)	774 (78.98)	45 (63.38)	0.002
Infection in last year				
Yes	171 (73.71)	149 (72.33)	22 (84.62)	1.80 (1)
No	61 (26.29)	57 (27.67)	4 (15.38)	0.18
Lower limb swelling				
Yes	426 (40.57)	367 (37.49)	59 (83.10)	57.12 (1)
No	624 (59.43)	612 (62.51)	12 (16.90)	<0.001
Upper limb swelling				
Yes	628 (59.81)	615 (62.82)	13 (18.31)	54.56 (1)
No	422 (40.19)	364 (37.18)	58 (81.69)	<0.001

^a^ Fisher's exact test.

LE, lymphedema.

The treatment categories of the patients are given in Table 3. The majority of patients did not get any treatment or advice before (66.4%). The most common treatment was exercise (50%), skin care advice (47%), massage (43%), and compression garment (40%). Psychological support was neglected in 91% of patients. A great number of patients expressed that their swelling was not under control. In Turkey the complex decongestive therapy is free in government hospitals for patients with obligative health insurance. Therefore the majority of the attendants replied to this question that treatment was free. But the cost of bandages for multilayer short stretch bandaging are not reimbursed and the amount of compression garments are only partially reimbursed in Turkey. Nearly 40% of the patients stated that if the treatment was not free, they could not cover the expenses for the treatment. Most of the patients suggested that lymphedema treatment was available for free within a reasonable travelling distance, as they are living in big cities or metropoles, but more than one third of them declared that the distance would prevent patients from accessing specialized centers. As the majority of patients did not have wounds or complicated lymphedema, they were not related to discharge from hospital or long stays in care centers (Table 4).

**Table 3.** The Treatment Categories (*N* = 051)

	*All patient (*N* = 1051),* n *(%)*	*Female (*n* = 980),* n *(%)*	*Male (*n* = 71),* n *(%)*	*χ^2^ (df) or* p*-value*
No treatment offered				
No	698 (66.41)	652 (66.53)	46 (64.79)	0.09 (1)
Yes	353 (33.59)	328 (33.47)	12 (35.21)	0.76
Skin care advice				
No	552 (52.52)	516 52.65)	36 (50.70)	0.10 (1)
Yes	499 (47.48)	464 (47.35)	35 (49.30)	0.75
Wound dressing				
No	1020 (97.05)	957 (97.65)	63 (88.73)	18.40 (1)
Yes	31 (2.95)	23 (2.35)	8 (11.27)	<0.001
Antibiotic				
No	934 (88.87)	880 (89.80)	54 (76.06)	12.63 (1)
Yes	117 (11.13)	100 (10.20)	17 (23.94)	<0.001
Massage				
No	594 (56.52)	553 (56.43)	41 (57.57)	0.04 (1)
Yes	457 (43.48)	427 (43.57)	30 (42.25)	0.83
Physiotherapy				
No	963 (91.63)	902 (92.04)	61 (85.92)	3.24 (1)
Yes	88 (8.37)	78 (7.96)	10 (14.08)	0.07
Compression garment				
No	638 (60.70)	599 (61.12)	39 (54.93)	1.06 (1)
Yes	413 (39.30)	381 (38.88)	32 (45.07)	0.30
Multilayer bandage				
No	716 (68.13)	669 (68.27)	47 (66.20)	0.13 (1)
Yes	335 (31.87)	311 (31.73)	24 (33.80)	0.72
Pneumatic compression pumps				
No	903 (85.92)	846 (86.33)	57 (80.28)	2.00 (1)
Yes	148 (14.08)	134 (13.67)	14 (19.72)	0.16
Debulking—lipedema—lymphatic surgery				
No	1042 (99.14)	971 (99.08)	71 (100.00)	
Yes	9 (0.86)	9 (0.92)	0 (0)	0.99^a^
Exercise advice				
No	517 (49.19)	479 (48.88)	38 (53.52)	0.57 (1)
Yes	534 (50.81)	501 (51.12)	33 (46.48)	0.45
Cellulitis advice				
No	811 (77.16)	761 (77.65)	50 (70.42)	1.96 (1)
Yes	240 (22.84)	219 (22.35)	21 (29.58)	0.16
Psychological support				
No	963 (91.63)	900 (91.84)	63 (88.73)	0.83 (1)
Yes	88 (8.37)	80 (8.16)	8 (11.27)	0.36
Complex decongestive therapy				
No	1035 (98.48)	965 (98.47)	70 (98.59)	0.99^a^
Yes	16 (1.52)	15 (1.53)	1 (1.41)	
Control of swelling				
No	414 (51.30)	372 (49.80)	42 (70.00)	9.07 (1)
Yes	393 (48.70)	375 (50.20)	18 (30.00)	0.003

^a^ Fisher's exact test.

**Table 4.** The Questions Related to Access, Distance, and Patient Costs of Treatment (*n* = 009)

	*Total (%)*	*Female (%)*	*Male (%)*	*χ^2^ (df) or* p*-value*
(1) Are patients' entire treatment complex decongestive therapy free?				
Yes	779 (77.21)	730 (77.49)	49 (73.13)	0.68 (1)
No	230 (22.79)	212 (22.51)	18 (26.87)	0.41
(2) If it is not free, could the patient cover the treatment expenses?				
Yes	538 (61.14)	504 (61.61)	34 (54.84)	1.11 (1)
No	342 (38.86)	314 (38.39)	28 (45.16)	0.29
(3) Is lymphedema treatment available in a reasonable distance?				
Yes	785 (77.95)	753 (80.02)	32 (48.48)	35.69 (1)
No	222 (22.05)	188 (19.98)	34 (51.52)	<0.001
(4) Does the distance prevent the patient from accessing a specialist center?				
Yes	302 (36.39)	276 (35.84)	26 (43.33)	1.35 (1)
No	528 (63.61)	494 (64.16)	34 (56.67)	0.25
(5) Do patients' lymphedema/wounds prevent discharge from hospital?				
Yes	47 (10.26)	35 (8.47)	12 (26.67)	14.58 (1)
No	411 (89.74)	378 (91.53)	33 (73.33)	<0.001
(6) Is the patient's lymphedema/wound the main reason for remaining in long-term care?				
Yes	79 (17.59)	63 (15.40)	16 (40.00)	15.20 (1)
No	370 (82.41)	346 (84.60)	24 (60.00)	<0.001
(7) Is the patient's lymphedema/wound the main reason for remaining in long-term home care?				
Yes	50 (11.47)	40 (10.05)	10 (26.32)	9.04 (1)
No	386 (88.53)	358 (89.95)	28 (73.68)	0.003

According to the demographics in the module data, most patients were in the age range of 45–64 years and were living with their partners or relatives. Eighty percent of the patients were owner occupiers and 55% had their own vehicle. As most of the patients were housewives, they were not the main provider for their family. Fifty-two percent of the attendants had primary school education with only 20% having a university diploma. As most patients were housewives (55%), they (92%) did not have to change or stop their job/work that would affect their family income (Table 5).

**Table 5.** Demographic Characteristics (*n* = 048)

	n *(%)*
Living with	
No one/live alone	109 (10.4)
Partner/spouse	724 (69.1)
Other relative	212 (20.2)
Friend	2 (0.2)
Other	1 (0.1)
Living accommodation	
Owner occupier	838 (80)
Public rented	11 (1.1)
Privately rented	189 (18)
Nursing home	5 (0.5)
Hospital	1 (0.1)
Supported living accommodation	4 (0.4)
Patients who had a car or other vehicle	
Yes	582 (55.5)
No	466 (44.5)
Working/job	
Employed full time	148 (14.1)
Employed part time	25 (2.4)
Retired	208 (19.8)
Unemployed looking for work	20 (1.9)
Not working because of illness	68 (6.5)
Looking after the house (housewives)	581 (55.4)
Full or part-time education or training	15 (1.4)
Other	3 (0.3)
Patients were the main provider	
Yes	202 (19.3)
No	846 (80.7)
Age of graduation, mean ± SD, median (minimum to maximum)	16.3 ± 4.9, 17 (8–45)
Degree of graduation	
None (elementary school)	549 (52.4)
School certificate/diploma	265 (25.3)
University diploma/degree	215 (20.5)
Master's degree	9 (0.9)
Doctorate	10 (1)
Patients who had to change their job or education/training	
Yes	52 (5)
No	996 (95)
Patients who had to stop work or education/training	
Yes	82 (7.8)
No	966 (92.2)
Patients who had been affected/reduced their family income because of the swelling	
Yes	99 (9.5)
No	949 (90.6)

SD, standard deviation.

The details of swelling in the module data are given in Table 6. The site of swelling was the upper extremity (arms) in 59% followed by legs (39.8%), Overall 55% did not have pitting edema. Tissues in the swollen area were mostly soft with 41% having a significant shape distortion. Thirty-six percent of the patients had not been told the reason for the swelling. Stemmer sign was positive in 75% of patients with lower extremity and 49% of patients in upper extremity swelling. The majority had ISL grade 2 swelling (61.7%) and 27.5% had ISL grade 1 lymphedema. The disability and QoL scores are given in Table 7. Most of the patients had impaired QoL and decreased functionality. These were prominent especially in lower limb chronic edema patients.

**Table 6.** Details of Swelling (*n* = 050)

	n *(%)*
Pitting	
Yes	475 (45.2)
No	575 (54.8)
Tissue in swollen area	
Soft	715 (68.1)
Hard	335 (31.9)
Shape distortion in the affected limb	
Yes	436 (41.5)
No	614 (58.5)
Patients who had been told the reason for the swelling	
Yes	671 (63.9)
No	379 (36.1)
The site of swelling	
Arm	617 (59.0)
Leg	416 (39.8)
Both	12 (1.2)
Stemmers sign	
Hand—positive	313 (49.3)
Foot—positive	329 (75.8)
Severity of the swelling	
ISL stage I	289 (27.5)
ISL stage II	648 (61.7)
ISL stage III	113 (10.8)

ISL, International Society of Lymphology.

**Table 7.** The Disability and Quality-Of-Life Scores (*n* = 050)

	*Mean* ± *SD*	*Median*	*Minimum to maximum*
WHODAS overall scores *N* = 1050	31.7 ± 21.8	27.1	0–100
EQ-5D scores (*n* = 1050)	0.56 ± 0.32	0.62	0.59–1
Overall health scores (*n* = 1050)	61.2 ± 20.5	60	0–100
LYMQOL—upper extremity (*n* = 630)			
Function	17.5 ± 6.1	16	10–40
Appearance	9.4 ± 3.6	9	5–20
Symptoms	12.3 ± 4	12	6–24
Emotion	11.3 ± 4.2	11	6–24
Overall	6.6 ± 1.8	7	0–10
LYMQOL—lower extremity (*n* = 429)			
Function	19.1 ± 6.4	19	8–32
Appearance	17.5 ± 5.9	17	7–28
Symptoms	11.8 ± 3.9	11	5–20
Emotion	12.9 ± 4.5	12	6–24
Overall	5.1 ± 2.1	5	0–10

EQ-5D, European Quality of Life Five Dimensional Questionnaire; LYMQOL, lymphedema quality of life; WHODAS, World Health Organization Disability Assessment Schedule.

The cancer data showed the majority (84%) had breast cancer followed by endometrium (8%), cervix (2.8%), and ovarian (2.4%) cancers. All but five of them had received treatment for cancer, with 53% having local cancer and 32% being in remission. The most common type of cancer treatment was surgery followed by chemotherapy and radiation therapy. The duration of lymphedema was <5 years in the majority of the patients (88.8%). Twenty-one percent of patients developed swelling within 3 months, 32% in 3–11 months, and 34% developed in 1–5 years after cancer treatment. The summary of cancer data is given in Table 8.

**Table 8.** Details of Cancer (*n* = 15)

	N *(%)*
Patients who had treatment for cancer	
Yes	710
No	5
Duration between swelling and cancer treatment (How long after the cancer treatment did you develop swelling in the affected area?)	
<3 Months	152 (21.3)
3–11 Months	234 (32.7)
1–5 Years	249 (34.8)
6–9 Years	40 (5.6)
10+ Years	32 (4.5)
Unknown	5 (0.7)
Not applicable	3 (0.4)
Current cancer status	
Cured/remission	229 (32.0)
Local cancer	383 (53.6)
Distant metastases	56 (7.8)
Do not know	47 (6.6)
Type of cancer	
Bladder cancer	2 (0.3)
Breast cancer	603 (84.3)
Cervical cancer	20 (2.8)
Colorectal cancer	2 (0.3)
Endometrial cancer	57 (8.0)
Head and neck cancer	1 (0.1)
Melanoma cancer	6 (0.8)
Ovarian cancer	17 (2.4)
Vulval cancer	2 (0.3)
Other cancer	16 (2.2)
Type of cancer treatments	
Surgery	703 (98.3)
Radiation therapy	569 (79.6)
Chemotherapy	595 (83.2)
Hormone therapy	306 (42.8)
Molecular target therapy	16 (2.2)
Other	1 (0.1)

The majority of patients (98%) did not have a wound. Of the patients with wounds, 55% had one to two wounds mostly grade 2 small venous ulcers with low exudate located in the legs. Most patients looked after their own wounds followed by physicians and hospital nurses. Nearly half of the wounds did not have signs of infection (47%) and had been present <6 months in the majority of the patients (70%). The wound details are given in Table 9.

**Table 9.** Details of Wounds (*n* = 1)

	N *(%)*
Provider wound care	
Physician	13 (61.9)
Podiatrist	0
Self-care	14 (66.7)
Family/friend	6 (28.6)
Hospital nurse	11 (52.4)
Practice nurse	0
Care home nurse	2 (9.5)
Wound care specialist nurse	0
Home care/community nurse	1 (4.8)
Lymphedema specialist nurse/therapist	1 (4.8)
Other	0
No. of wounds	
One	7 (33.3)
Two	9 (42.9)
Three	2 (9.5)
Four	1 (4.8)
Five	1 (4.8)
Six	1 (4.8)
Missing	1 (4.8)
Pressure ulcer	
None	13
Grade 1	0
Grade 2	5 (62.5)
Grade 3	2 (25.0)
Grade 4	1 (12.5)
Leg/foot ulcer cause	
No leg/foot ulcer	6
Venous ulcer	6 (40.0)
Arterial ulcer	1 (6.7)
Mixed (venous/arterial)	1 (6.7)
Neuropathic	1 (6.7)
Neuroischemic	0
Other foot ulcer	2 (13.3)
Do not know ulcer type	7 (46.7)
Acute/surgical wound	
No acute/surgical wound	10
Primary closure	0
Open surgical wound	0
Postsurgical breakdown	2 (18.2)
Dehisced wound	5 (45.5)
Traumatic wound	2 (18.2)
Do not know wound type	2 (18.2)
Exudate level	
None	9 (42.9)
Low	7 (33.3)
Medium	4 (19.1)
High	1 (4.8)
Location of the wounds	
Head or neck	21 (100)
Arms	21 (100)
Chest	19 (90.5)
Abdomen	21 (100)
Back	20 (95.2)
Sacrum	21 (100)
Hips	21 (100)
Upper leg	20 (95.2)
Groin	21 (100)
Lower leg/ankle	9 (42.9)
Foot	14 (66.7)
Other	20 (95.2)
Wound area	
Small: <10 cm^2^	17 (81)
Medium: >10 and <25 cm^2^	4 (19.1)
Large: >25 cm^2^	0
Closed surgical wound	0
Not applicable	0
Wound duration	
Primary	16 (76.2)
Recurrent	5 (23.8)
Time/duration of wounds	
<1 Week	0
1–2 Weeks	2 (9.5)
2–4 Weeks	2 (9.5)
4–6 Weeks	3 (14.3)
6 Weeks to <3 months	4 (19.1)
3 Months to <6 months	3 (14.3)
6 Months to <1 year	1 (4.8)
1 Year to <5 years	3 (14.3)
5 Years or more	1 (4.8)
Do not know	2 (9.5)
Wound infection	
Yes	9 (42.9)
No	10 (47.6)
Unknown	2 (9.5)
Frequency of dressing change	
Twice daily	6 (28.6)
Daily	6 (28.6)
Alternate days	3 (14.3)
Two to three times per week	5 (23.8)
Once a week	1 (4.8)
Other	0

One of the fundamental aims of the ILF is to support countries in the development of data to establish the size of the problem of chronic edema. Such data are essential in supporting the introduction of evidence-based practice and enabling each national framework to argue for appropriate financing and reimbursement.^17^ According to the aims of LIMPRINT as an international epidemiological research study; the preliminary demographic results of this study provided evidence-based data for the demographic and clinical properties of Turkish lymphedema patients. The LIMPRINT study brought a great opportunity and vision to our community. The reimbursement of pressure garments was very low in grades 2 and 3 lymphedema patients in Turkey. The ALA have prepared a file for reimbursement of care about the condition and impact of lymphedema based on the results of the LIMPRINT-Turkey study. As a partner of the ILF and of the LIMPRINT study, ALA have summarized and indicated the characteristics of Turkish lymphedema patients and demonstrated their efforts for increasing the awareness and collaboration between health professionals on a national basis. The Turkish Social Security Institution has had meetings with ALA members, made rectification, and taken the decision to pay more for the reimbursement of pressure garments for lymphedema patients with grades 2 and 3 lymphedema. We believe that the final data indicate not only the size of the problem but also the impact of chronic edema on patient lives in terms of functionality and QoL. This will assist lymphedema services to provide evidence-based care. The Turkish LIMPRINT study results demonstrate that many patients cannot access treatment services because of the distance and cannot afford to pay for costly treatments as these are not completely reimbursed. We hope this evidence-based data will change the national policies for the care of Turkish patients with lymphedema or chronic edema.

## Conclusion

This final LIMPRINT data reflect that upper extremity lymphedema is more common than lower extremity and the major cause is cancer treatment, predominantly breast cancer in the Turkish LIMPRINT. The most striking results are that the patients suffer for a long time, most of the patients have uncontrolled lymphedema mostly grade 2 and have not received any previous treatment before the study. Turkish patients had less wounds compared with other studies undertaken in the LIMPRINT study. This is most certainly because of the center characteristics that were rehabilitation services treating a high proportion of cancer patients, particularly breast cancer, rather than dermatology or vascular surgery services. The majority of patients had reduced functional status and decreased QoL. Although most of the patients had social health security for free complex decongestive therapy treatment, their ability to access these centers was more difficult than previously estimated. National health policies and planning are needed for the prevention, diagnosis, and treatment of those suffering this neglected condition in Turkish patients.
